# Case Report: Primary Pulmonary Angiosarcoma With Brain Metastasis

**DOI:** 10.3389/fbioe.2021.803868

**Published:** 2022-01-07

**Authors:** Xiangjun Tang, Jing Zhu, Fangcheng Zhu, Hanjun Tu, Aiping Deng, Junti Lu, Minghuan Yang, Longjun Dai, Kuanming Huang, Li Zhang

**Affiliations:** ^1^ Department of Neurosurgery, Taihe Hospital, Hubei University of Medicine, Shiyan, China; ^2^ Department of Respiratory and Critical Care Medicine, The Central Hospital of Wuhan, Tongji Medical College, Huazhong University of Science and Technology, Wuhan, China; ^3^ School of Basic Medical Sciences, Hubei University of Medicine, Shiyan, China

**Keywords:** primary pulmonary angiosarcoma, brain metastasis, autopsy, diagnose, treatment

## Abstract

Primary pulmonary angiosarcoma (PPA) is a rare malignant vascular tumor, of which early diagnosis is challenging due to lack of specific clinical manifestations and a low level of suspicion. Here, we report a case of PPA presented with advanced brain metastasis. A 21-year-old patient with 1 week history of headache and mild cough was hospitalized for a head injury. Head MRI showed multiple intracranial lesions with brain edema. Chest CT displayed bilateral pulmonary infiltrates with mediastinal lymph node enlargement. After 2 months of anti-tuberculosis treatment, the patient was readmitted for persistent headache and cough with occasional hemosputum along with worsening pulmonary and intracranial lesions. Despite seizure prophylaxis and control of intracranial pressure and brain edema, his symptoms progressively aggravated, accompanied by cough with bloody sputum, frequent epileptic seizures, and hypotension. He eventually developed coma and died within 3 months of onset of symptoms. An autopsy confirmed PPA with brain metastasis.

## Introduction

Angiosarcoma is an uncommon malignant endothelial tumor originated from blood or lymphatic vessels, accounting for about 2% of all soft tissue sarcomas. The tumor can arise in any organ, most often in the head and neck cutaneous tissue (Young et al., 2010). It is aggressive and often multicentric, with a high rate of local recurrence and distant metastasis associated with an overall poor prognosis. The lung is usually the major metastatic site of angiosarcoma, while primary pulmonary angiosarcoma (PPA) is extremely rare (Chen et al., 2010; ObesoCarillo et al., 2013). Patients with PPA may present with various degree of respiratory symptoms, however, definite diagnosis is difficult to make based on imaging or bronchoscopy, and often requires biopsy through surgical resection. We report here an uncommon case of PPA presented with advanced brain metastasis.

## Case Report

The patient was a 21-year-old male non-smoker without significant past medical history other than alcohol drinking (150 g/day). One week before admission, the patient developed a swelling headache without obvious inducement. The headache progressively intensified, accompanied by minor coughs without hemoptysis or other neurological symptoms. Two days before admission, the patient was admitted to a local hospital for head trauma and found to have multiple intracranial space-occupying lesions on a head CT scan. He was transferred to our hospital for further diagnosis and treatment.

**FIGURE 1:**
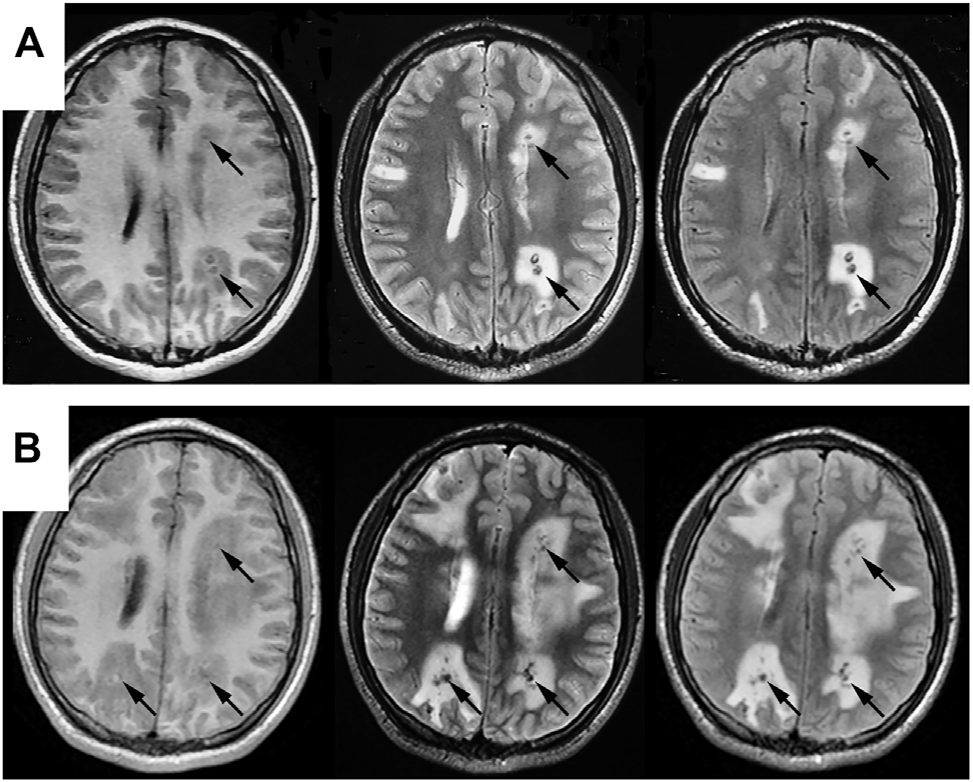
Comparisons of cranial MRI results before **(A)** and after **(B)** admission. Both **(A**,**B)** reveal multiple nodular abnormal signals in bilateral cerebral and cerebellar hemispheres, mainly in the cortical and cortical medullary junctions, with lesions series **(B)** significantly larger than that in series **(A)**. T2WI and T2flair are mainly low signals, with high signals in some lesions. T1flair shows equal and low signals, with obvious edema around the lesions.

**FIGURE 2:**
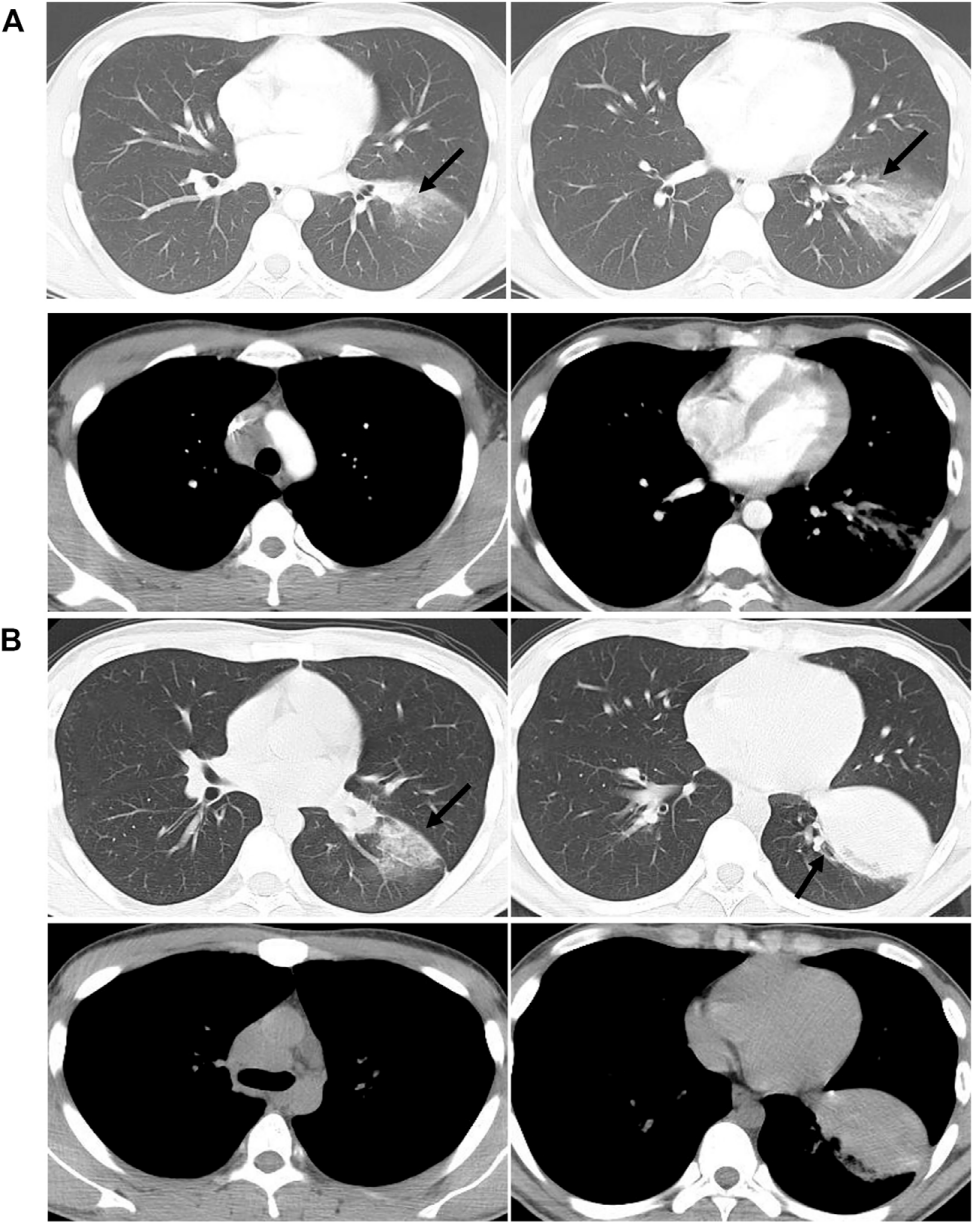
Comparisons of chest CT results before **(A)** and after **(B)** admission. **(A)** shows the patchy shadow in the left lower lobe, with mediastinal lymphadenopathy. **(B)** shows that the left lower lung lesions are enlarged than before, with a solid shadow and lymphadenopathy.

On admission, the patient’s vital signs were stable, mental status normal and physical examinations unremarkable. Laboratory tests showed WBC 11.42 × 10^9^/L, neutrophil 8.18 × 10^9^/L and eosinophil 0.51 × 10^9^/L, normal coagulation tests, normal tumor markers (CEA and AFP), and negative PPD test. Plain and enhanced head MRI revealed bilateral multiple intracranial lesions suggesting tuberculosis or possible metastatic tumors (Figure 1A). An enhanced CT scan of the chest showed bilateral pulmonary patchy infiltrates, most prominent in the left lower lobe dorsal segment, and an enlarged mediastinal lymph node (about 2.5 × 1.6 cm) without pleural effusion (Figure 2A). The patient was treated with intracranial pressure lowering agents and seizure prophylaxis as well as anti-tuberculosis agents and other antibiotics. One month later, patient’s headache and cough remained unchanged, while repeat CT scan showed that both intracranial and pulmonary lesions had progressed significantly. The patient then underwent a bronchoscopy, which displayed bronchial inflammation and hemorrhage in the left lower lobe basal segment, as were consistent with bronchial biopsy results. No cancer cells were detected in the lavage. Considering the duration of treatment, the patient was advised to continue anti-tuberculosis and seizures prophylaxis as an outpatient.

**FIGURE 3:**
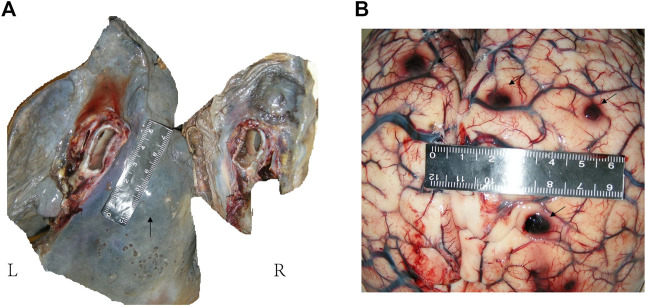
Anatomical examination of lung and brain. **(A)**: Gray-white hard nodules were seen in the hilar and lower lobe. There was a hard mass in the mediastinum, about 5 cm × 2 cm in size. A small amount of gelatinous material was seen in the right main bronchus and pulmonary congestion was seen on section. **(B)**: Multiple hard nodules with bleeding were seen on both sides of the cerebral hemisphere and cerebellum.

**FIGURE 4:**
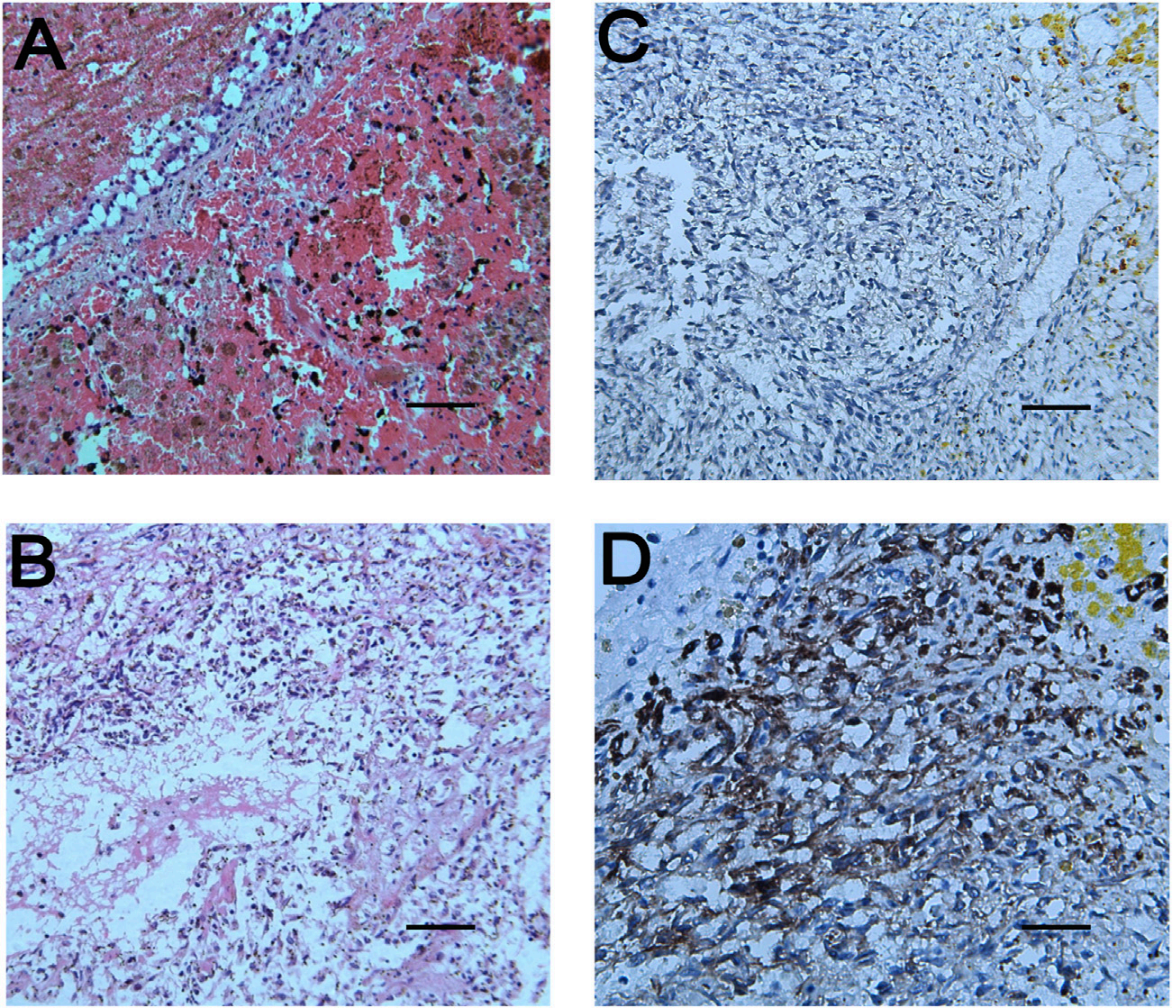
Pathological results of lung and brain tissues. **(A)**: The tumor cells in the pulmonary nodules are arranged in a single thin-walled vascular lumen, filled with red blood cells and accompanied by necrosis, and have a large nuclear-cytoplasmic ratio, dark-colored nuclei and atypia. **(B)**: The lesions of the brain tissue are similar to those of the pulmonary nodules, accompanied by hemorrhage and necrosis. The surrounding brain tissue can be seen in the cuff-like infiltration of lymphocytes-based inflammatory cells around the blood vessels. Necrophages and satellite cells can also be observed. **(C**,**D)** respectively represent immunoreactivity for CD31 and CD34 on immunohistochemical examination (Magnifications: **(A)**, ×100; **(B**,**C)**, ×200; **(D)**, ×400).

The patient continued suffering from intermittent headache, cough with phlegm and occasional hemosputum. When he returned for follow-up 1 month later, his head MRI showed bilateral lesions wider than those of the previous one (Figure 1B). Chest CT indicated bilateral scattered lesions, with the lesion in the left lower lobe and the mediastinal lymph node larger than before and associated with high density lesions in the lower left oblique fissure (Figure 2B). Additionally, spinal tap was performed, and examination of cerebrospinal fluid revealed colorless, transparent fluid without clots, negative for Pan’s test, total protein 0.55 g/L, glucose 4.12 mmol/L, and no cells or acid-fast bacilli found. While the patient continued to receive treatment for brain edema, anti-infection and seizure prophylaxis, his condition deteriorated progressively with worsening headache, frequent convulsions, hypotension, leading to coma and death. With the consent of the patient’s family, an autopsy was performed, and it revealed multiple nodular lesions in the lungs and brain (Figure 3), no tumor in other organs. Immunohistochemistry of the lungs and brain displayed CK (-), Ki-67 (-), CD31 (+), CD34 (+), EMA (-) and GFAP (-). Histopathology of the lesions confirmed the diagnosis of PPA with brain metastasis (Figure 4).

## Discussion

PPA is a malignant tumor originating from pulmonary vascular endothelial cells and is a type of deep soft tissue angiosarcoma. The occurrence of the disease is extremely rare (Maglaras et al., 2004; Campione et al., 2009; Chen et al., 2010; ObesoCarillo et al., 2013). The average age of onset is 47.9 years-old (ranging 2–79 years) with occurrences more common in men (Drosos et al., 2019). The clinical manifestations of PPA are not specific. The most common symptoms of the disease reported so far are hemoptysis or hemosputum, cough, dyspnea, chest pain, and other non-specific symptoms such as fatigue, weight loss and fever (Ren et al., 2016). However, some patients may be asymptomatic during the course of the disease. In this case, the patient was younger than most reported patients (Drosos et al., 2019; Virarkar et al., 2019), and showed only minor coughs with occasional hemosputum, while headache due to metastasis was prominent. These factors contributed the misdiagnosis as tuberculosis.

Imaging is important in the diagnosis of pulmonary angiosarcoma, however, chest X-ray usually fails to detect fine changes of pulmonary angiosarcoma. PPA may present as multifocal or solitary nodular lesions on CT scans, with or without ground-glass changes, or with solidity changes (Ozcelik et al., 2006; Chen et al., 2010; Weissferdt and Moran, 2010). It is essential to differentiate pulmonary angiosarcoma from infectious diseases such as pneumonia and tuberculosis. Atasoy *et al.* reported a solitary PPA with large soft tissue masses in the left upper lobe, with mediastinal invasion and left parabronchial lymph node enlargement on the CT (Atasoy et al., 2001). At admission, our patient had pulmonary CT findings of patches in the dorsal segment of the left lower lobe, associated with ground-glass changes and enlargement of mediastinal lymph nodes, as well as multiple intracranial nodules on the cranial MRI. On the other hand, laboratory test suggested presence of infection, and bronchoscopy showed no intratracheal space-occupying lesion (mass effect), pointing to infectious nature and leading to our initial misdiagnosis. However, when both pulmonary and intracranial lesions progressed rapidly under anti-tuberculosis treatment, with chest CT scan showing scattered nodules with ground-glass opacity and some solid masses with uneven density, attention should have been directed to the possibility of the highly invasive lung malignancy.

PPA accounts for 9.3% of total angiosarcomas (Drosos et al., 2019) and tends to grow latently. While local invasion and blood metastasis have often occurred at the time of detection (Maglaras et al., 2004; Grafino et al., 2016), PPA with brain metastasis was extremely rare. Our patient has this feature, with multiple intracranial metastases at the time of presentation. The main lethal factors of common brain metastatic tumors are brain edema and space-occupying effect, while our patient died of uncontrollable epilepsy and cerebral edema (Kim et al., 2015; Sun et al., 2015).

Lung biopsy and immunohistochemistry are the gold standards for the diagnosis of pulmonary angiosarcoma. Percutaneous fine needle aspiration cytology, bronchoscopic lung biopsy and thoracotomy/thoracoscopic biopsy are important means to obtain tissue specimens (Kojima et al., 2003; Campione et al., 2009; Wan Musa et al., 2010). Pathological diagnosis has also been reported through autopsy. Among them, bronchoscopy lung biopsy has a low positive rate for diagnosis of PPA, mainly because the small amount of biopsy tissue may not be sufficient to contain typical pathological features, and involvement of bronchi is not common, as were the case in our patient. Therefore, open lung biopsy is a more effective diagnostic method. Abnormal pleomorphic malignant endothelial cells are characteristic of angiosarcoma. These cells can be round, polygonal or spindle-shaped with or without epithelioid changes. In well-differentiated areas, abnormal endothelial cells form sinuses that connect to normal blood vessels. In invasive lesions, this structure becomes chaotic and does not form a well-defined cavity. In poorly differentiated regions, malignant endothelial cells form a continuous monolayer, often accompanied by epithelioid morphology. Although cytological atypia exists, the tumor cells are mainly characterized by relatively single, mitosis, necrosis and hemorrhage (Keel et al., 1999), which is consistent with the autopsy results of our patient. Immunohistochemical markers for diagnosis of pulmonary angiosarcoma include factor VIII-related antigen, CD34 and CD31, with factor VIII-related antigen being the most specific but not as sensitive as CD31, which is expressed in 90% of angiosarcomas (Huo et al., 2006). Our patient progressed rapidly and die of brain lesions scattered in multiple foci with hemorrhage, necrosis and edema. Positive CD34 and CD31 and histopathology on autopsy tissues confirmed the final diagnosis.

At present there is no unified treatment regimen for PPA. Surgical resection, radiotherapy, chemotherapy, immunotherapy and combined radiotherapy and chemotherapy have been reported in the literature (Kojima et al., 2003; Wilson et al., 2008; Chen et al., 2010; Grafino et al., 2016; Shirey et al., 2018). No treatment has been clinically proven to be effective, but treatment under certain conditions may be effective, such as early removal of focal lesions. However, the disease is often diagnosed at a late stage and beyond the indications for surgery. It has been reported that recombinant IL-2 immunotherapy combined with radiotherapy or gemcitabine combined with docetaxel may also be effective (Kojima et al., 2003). The current literature reports that the prognosis of PPA is extremely poor, with median survival time 7 months in patients with a single lesion, and 2 months in those with multiple lesions (Ren et al., 2016). The disease in this case developed rapidly and was quite aggressive, with advanced lesions beyond surgically resection at presentation, resulting in death in less than 3 months. Therefore, early detection and identification is crucial.

In conclusion, PPA is an extremely rare malignant tumor with a very poor prognosis. Because of the lack of specific clinical manifestations, timely diagnosis is difficult. The PPA patient reported here presented with early and advanced brain metastasis and was diagnosed after autopsy using histopathology and immunohistochemistry biomarkers. This case cautions us that PPA should be considered when a patient’s MRI shows multiple mixed signals accompanied by obvious brain edema of unknown origin. Once the disease is suspected, biopsy combined with immunohistochemistry should be used to confirm the diagnosis as soon as possible.

## Data Availability

The raw data supporting the conclusions of this article will be made available by the authors, without undue reservation.
